# Working in the Neonatal Intensive Care Unit Is More Than Saving a Baby’s Life

**DOI:** 10.4269/ajtmh.22-0293

**Published:** 2022-08-29

**Authors:** Godbless M. Philipo, Hannah Lee

**Affiliations:** ^1^ Arusha Lutheran Medical Centre, Pediatrics, Arusha, Tanzania;; ^2^ University of Minnesota, Internal Medicine/Pediatrics, Minneapolis, Minnesota

Thirty-two-week-old Maasai twins were brought to Selian Hospital in Arusha, Tanzania, with evidence of apparent twin–twin transfusion syndrome. They were delivered at home and then taken to a nearby dispensary health facility that frequently does not have a staff doctor present. They were then referred to us. One twin (twin 1) was doing well with minimal oxygen support. However, it became evident that twin 2 was struggling to breathe, with oxygen saturation levels in the 70s, and needed increased support. At Selian, patients must pay for services before they receive any laboratory work, imaging, or oxygen therapy. This was instituted because, historically, there have been difficulties with patients paying their bill, and it is a preventive measure for keeping the hospital afloat. However, because the majority of Selian patients do not have health insurance, things that may be seemingly easy fixes for symptoms, become difficult for patients of all sizes and ages to obtain.

As the pediatric registrar on service, I oversaw taking care of the two babies admitted to the neonatal intensive care unit. I was post-call and had been up the entire previous night by the time we were evaluating twin 2 in the afternoon. Our team consisted of nursing staff; an intern; a visiting chief resident doctor from the United States, Dr. Hannah Lee; and our supervising pediatric consultant, Dr. Theresia Laizer. With the three oxygen saturation monitors that were available, we were at bedside, switching the monitors every few minutes to determine whether there was a difference in the readings. Repeatedly, the same findings were demonstrated in ranges that we were not happy with 70%, barely 80%.

“This baby needs surfactant,” I said to Dr. Lee, the nurses, and the intern. However, I knew that none was available at Selian. Oxygen was started on both babies. Twin 2 was on bubble continuous positive airway pressure and the other one on low-flow nasal cannula. The oxygen cylinder was provided by the hospital only on an emergency basis and the family needed to pay to secure the cylinder, and to get an additional cylinder when that the first one expired. After several hours of monitoring, I finally decided I needed to go get some surfactant. Dr. Lee and the intern would continue to monitor the care of the infant while I was gone.

I drove 30 minutes to a hospital closer in town—Arusha Lutheran Medical Center (ALMC)—where I knew we could borrow some surfactant. I grabbed an intubation kit and the medication and drove back to Selian. I wanted to at least give the baby a chance to breathe, because I knew at other hospitals, these babies receive that chance. The pediatric consultant, Dr. Laizer, rallied and talked to the oxygen providers and obtained another cylinder of oxygen as the first one was about to be finished, and she promised the oxygen providers that the mother would pay them later. It was a difficult intubation, but with Dr. Lee bagging the infant throughout, and the intern monitoring vitals, our team delivered surfactant successfully after three attempts. The saturations slowly improved to the 90s. We also ordered a blood transfusion. At that point the baby was now breathing more comfortably, coupled with the extra oxygen cylinder that was lent to us with Dr. Laizer’s help. With the baby more stabilized, the team disbanded for the day, hoping for a quiet night for the two twins.

The next morning, our team found out that, unfortunately, twin 2 was not able to receive blood because the blood bank at Selian did not have the blood type compatible with the baby’s. Again we had to ask ALMC for help to determine whether there was any blood availability there. I went and collected the blood and returned with it to complete the transfusion. Despite the transfusion, twin 2 was still struggling, barely stable. He ended up needing another oxygen cylinder, which our team managed to obtain. However, overnight, the baby deteriorated; he became more hypoxic, despite continuous positive airway pressure and oxygen support. He died on day 3 despite the team’s best efforts.

As a pediatric registrar working at Selian Hospital through my rotations, a story like this of the twins is far too common. It has been reported that neonatal mortality in Tanzania has ranged from 26 to 40 deaths per 1,000 live births for the past three decades, even though the overall neonatal mortality rate is decreasing in Tanzania. Although the story of the twins is not a new one, we tried our best to give the babies at Selian a chance, because I know these babies would have survived if only they were born 30 minutes away—at ALMC. This situation drives me to do better, like driving 30 minutes to obtain surfactant and blood regardless of how tired I might be. I am aware of this difference in care, and aware of what *could* be done. With this knowledge, if we give our best efforts as a team, it helps me with my peace of mind and we can feel like we have done everything possible when I am talking to the mother. We all know there is no replacing this life and that this child deserved a chance at life. The fact that I can tell the mother we tried absolutely everything to save her baby’s life is important for all of us, because we all deserve a chance at life. In addition, in our society and culture in Tanzania—especially in the Maasai culture—when a woman gives birth and has a baby, she has essentially fulfilled her job in society. If a mother is not able to go home with her baby, the social welfare of the mother is going to be damaged, because the woman may become dispensable to the husband. Not only is there the emotional loss experienced by the mother, but also there are consequences in her marriage as well—and her social and economic status. With this awareness, we are compelled to put forth our best efforts to help all infants that come to the hospital. I know the system does not favor the babies, so if we do not attempt to go the extra mile, two lives are at stake: the mother’s and the infant’s.

